# Mobile locally operated detachable end-effector manipulator for endoscopic surgery

**DOI:** 10.1007/s11548-014-1062-4

**Published:** 2014-05-06

**Authors:** Toshikazu Kawai, Myongyu Shin, Yuji Nishizawa, Yuki Horise, Atsushi Nishikawa, Tatsuo Nakamura

**Affiliations:** 1 Graduate School of Engineering, Osaka Institute of Technology, 5-16-1 Omiya, Asahi Ward, Osaka , 535-8585 Japan; 2 Department of Colonic Surgery, National Cancer Center Hospital East, 6-5-1, Kashiwanoha, Kashiwa , 277-8577 Japan; 3 Department of Mechanical Science and Bioengineering, Graduate School of Engineering Science, Osaka University, 1-7 Machikaneyama, Toyonaka , 560-0043 Japan; 4 Textile Science and Technology, Shinshu University, 3-15-1, Tokida, Ueda , 386-8567 Japan; 5 Frontier Medical Sciences, Kyoto University, 53 Kawahara-cho, Shogoin, Sakyo-ku, Kyoto , 606-8507 Japan

**Keywords:** Local operation, Endoscopic surgery, Mobile surgical robot, Third arm, Disassembled mechanism

## Abstract

**Purpose:**

Local surgery is safer than remote surgery because emergencies can be more easily addressed. Although many locally operated surgical robots and devices have been developed, none can safely grasp organs and provide traction. A new manipulator with a detachable commercial forceps was developed that can act as a third arm for a surgeon situated in a sterile area near the patient. This mechanism can be disassembled into compact parts that enable mobile use.

**Methods:**

A mobile locally operated detachable end-effector manipulator (LODEM) was developed and tested. This device uses crank-slider and cable-rod mechanisms to achieve 5 degrees of freedom and an acting force of more than 5 N. The total mass is less than 15 kg. The positional accuracy and speed of the prototype device were evaluated while performing simulated in vivo surgery.

**Results:**

The accuracy of the mobile LODEM was 0.4 mm, sufficient for handling organs. The manipulator could be assembled and disassembled in 8 min, making it highly mobile. The manipulator could successfully handle the target organs with the required level of dexterity during an in vivo laparoscopic surgical procedure.

**Conclusions:**

A mobile LODEM was designed that allows minimally invasive robotically assisted endoscopic surgery by a surgeon working near the patient. This device is highly promising for robotic surgery applications.

## Introduction

Endoscopic surgery has become popular in recent years because it is a minimally invasive but accurate surgical procedure that offers an expanded field of view and leaves only small scars. When performing surgery in which it is important to preserve organ functions, the affected area must be identified and the field of view expanded safely and effectively. To perform accurate surgery, it is important for the organ to be grasped and pulled in two directions, and cut when it is under sufficient tension. However, surgeons must manipulate tools with insufficient degrees of freedom (DOFs) and the effects of hand tremors need to be minimized. In addition, cooperation is required between the doctor performing the surgery and assistants using an endoscope or forceps. Many master-slave controlled manipulators [1] have been developed to solve these issues. Well-known surgical robots in current clinical use are the ZEUS [2] and da Vinci [3] systems, which have three or four arms attached to a tool with 6 or 7 DOFs; they are operated remotely by a surgeon in a non-sterilized area. Such systems allow high positional accuracy because of the use of motion scaling and low-pass filtering to counteract tremors. Another master-slave controlled surgical robot in clinical use is the NeuRobot system [4, 5], which is used for neurosurgery.

Because of the possibility of emergencies occurring, local operation must be considered safer than remote operation. For this reason, our goal is robotically assisted surgery performed by a single surgeon situated in a sterile area near the patient. A large number of locally operated surgical robots and devices have been developed. The manually controlled mechanical forceps Radius [6] and Autonomy [7] have 3 DOFs. The passive brake controlled stabilizer used in the intelligent armrest EXPERT [8, 9] allows tremor elimination. The endoscope-holding robot AESOP [10] can be operated via voice control, Naviot [11] via forceps-mounted button control, and FreeHand [12] via head-mounted sensor control; these systems have 2 or 3 DOFs. These endoscope-holding robots eliminate the need for cooperation with an endoscope assistant. However, there is no locally operated forceps robot that can grasp organs and provide traction.

Except for university hospitals where interns or residents are educated, there are advantages to single surgeons performing endoscopic surgery without an endoscope assistant, in that the number of operations can be increased. This is particularly true for hospitals where a large number of surgeons are endoscope specialists. Even in hospitals where few surgeons are endoscope specialists, those specialists would often prefer to change their technique from open abdominal or chest surgery to endoscopic surgery, since the latter is minimally invasive to patients. Again, this could be accomplished more efficiently if an endoscopic assistant was not required. One problem with the well-known da Vinci system is that it is difficult for a single surgeon to operate alone in the clean area, both for safety reasons and because of the need for tool changing. However, if a locally operated forceps robot was developed, a surgeon could perform safe, accurate endoscopic surgery holding mechanical forceps in the hands stabilized by intelligent armrests, while controlling an endoscope-holding robot and the new forceps robot using a choice of interfaces. Because anastomosis of arteries with diameters of less than 1 mm has been successfully performed using the EXPERT system, such procedures are also expected to be possible by robotically assisted surgery performed by a single surgeon.

To this end, we propose a manipulator that can act as a third arm for a surgeon in the sterile environment of the operating room. It uses a detachable commercial forceps, allowing the surgeon to intuitively handle internal organs. It can be operated by the surgeon’s hand or foot and can be removed from the surgical table as required. For conventional manipulation using a pivot point at the abdominal wall, 5 DOFs are required: the roll, pitch and yaw axes, the insertion/extraction axis, and the open/close axis. It is also necessary for the grasp and traction force to be greater than 5 N [13]. We therefore developed a locally operated detachable end-effector manipulator (LODEM) based on a selective compliance assembly robot arm (SCARA) with 5 DOFs, an acting force of more than 5 N, and an accuracy of 0.5 mm, and applied it to it in vivo laparoscopic cholecystectomy [14, 15].

The compact and simple structure of the developed device allows its easy introduction into operation rooms. Simple transportation and installation of the manipulator are important because it allows the doctor to be mobile. It is therefore necessary to use a mechanism that can be disassembled into compact parts. However, although the SCARA LODEM [14] is compact, it uses a remote pivot point that is controlled mathematically, so that its operation is not intuitive. The parallel-link manipulator [16] offers high accuracy and is capable of operating in narrow working spaces, but it also has the same variable pivot point. The R-guided manipulator [17] uses a fixed mechanical pivot point, but it is difficult to disassemble into compact parts. To overcome these difficulties, we previously proposed crank-slider and cable-rod mechanisms [18]. The crank-slider mechanism converts linear motion into rotary motion and can have an ‘L’ shape or an ‘I’ shape. The cable rod is composed of an outer stainless steel tube and an inner stainless steel cable. The cable rod is connected to the actuator that is driven by a forceps and the forceps-driving mechanism. We also developed a mobile LODEM which can be disassembled into compact parts and we applied it to a simulated surgical procedure.

In the present study, the design details of the proposed mobile LODEM are introduced. In addition, the accuracy and speed of the prototype device are evaluated while performing simulated in vivo surgery.

## Materials and methods

### Design

**Fig. 1:**
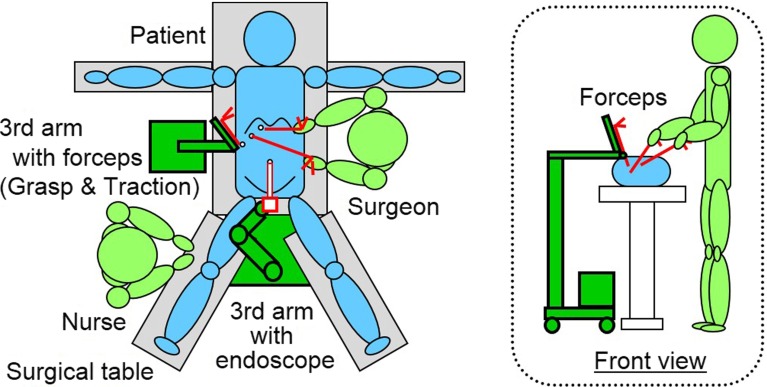
Concept of endoscopic surgery supported by locally operated detachable end-effector manipulator (LODEM). The manipulator is attached to a forceps and operated by a surgeon in the sterile area

The concept of robotically assisted endoscopic surgery performed by a surgeon is shown in Fig. 1. The mobile LODEM positioned on the right-hand side of the surgical table is attached to a forceps and acts as a third arm for the surgeon. The operator stands on the left-hand side of the table and controls the manipulator in a sterile area. The manipulator itself is draped with a sterile cover, and a separate sterilized attachment is used to connect the forceps to the manipulator.

**Fig. 2:**
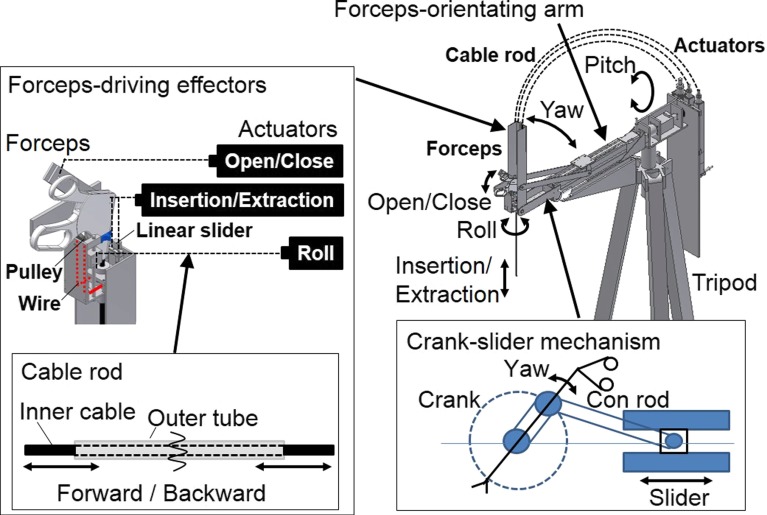
Design of mobile LODEM. The manipulator with 5 DOFs comprises a forceps-orienting arm using a crank-slider mechanism (*right panel*) and forceps-driving effectors using cable rods (*left panel*)

The mechanical design of the LODEM is shown in Fig. 2. The manipulator has 5 DOFs and comprises a forceps-orientating arm that uses a crank-slider mechanism, and forceps-driving effectors using cable rods. For safety reasons, electrical parts such as the actuators for driving these mechanisms can be placed far from the patient. The forceps-orienting arm has two axes: pitch and yaw. The pitch axis is driven by a harmonic geared motor. The yaw axis has a crank-slider mechanism driven by a ball screw motor. Two con rods connect the linear slider driven by the ball screw motor and a rotating crank. The pivot point is determined by these two axes. At the pivot point on the abdominal wall, the manipulator is enough large to cover the trocar, which is the guide for the forceps. The forceps-driving effectors have three axes: roll, insertion/extraction and open/close. The roll axis has a wire and pulley mechanism that transforms linear motion into rotary motion. The insertion/extraction axis uses a slider mechanism. The open/close axis has a fixed plate and a rotary plate mechanism that transforms linear motion into rotary motion. The hand grip part of the forceps is attached to these plates. These three axes are driven by ball screw actuators connected using cable rods. The opposite ends of the cable rods are connected to the forceps-driving mechanism for the wire in the case of the roll axis, the slider in the case of the insertion/extraction axis, and the rotary plate in the case of the open/close axis. Moving the actuator forward or backward is achieved by pushing or pulling the inner cable. For the insertion/extraction axis, a different cable rod is used for each direction, whereas for the other two axes, a single cable rod is used.

The manipulator can be disassembled into the main arm, the actuators, the cable rods and the tripod. The main arm can be changed from an ‘L’ shape to an ‘I’ shape by applying a force to its tip. For safety reasons, the position and orientation of all five axes are maintained even in the event of a power loss. The manipulator can be removed from the surgical table as required. To control the forceps attached to the manipulator, the operator uses a button controller attached to a handheld forceps.

### Prototype

**Fig. 3:**
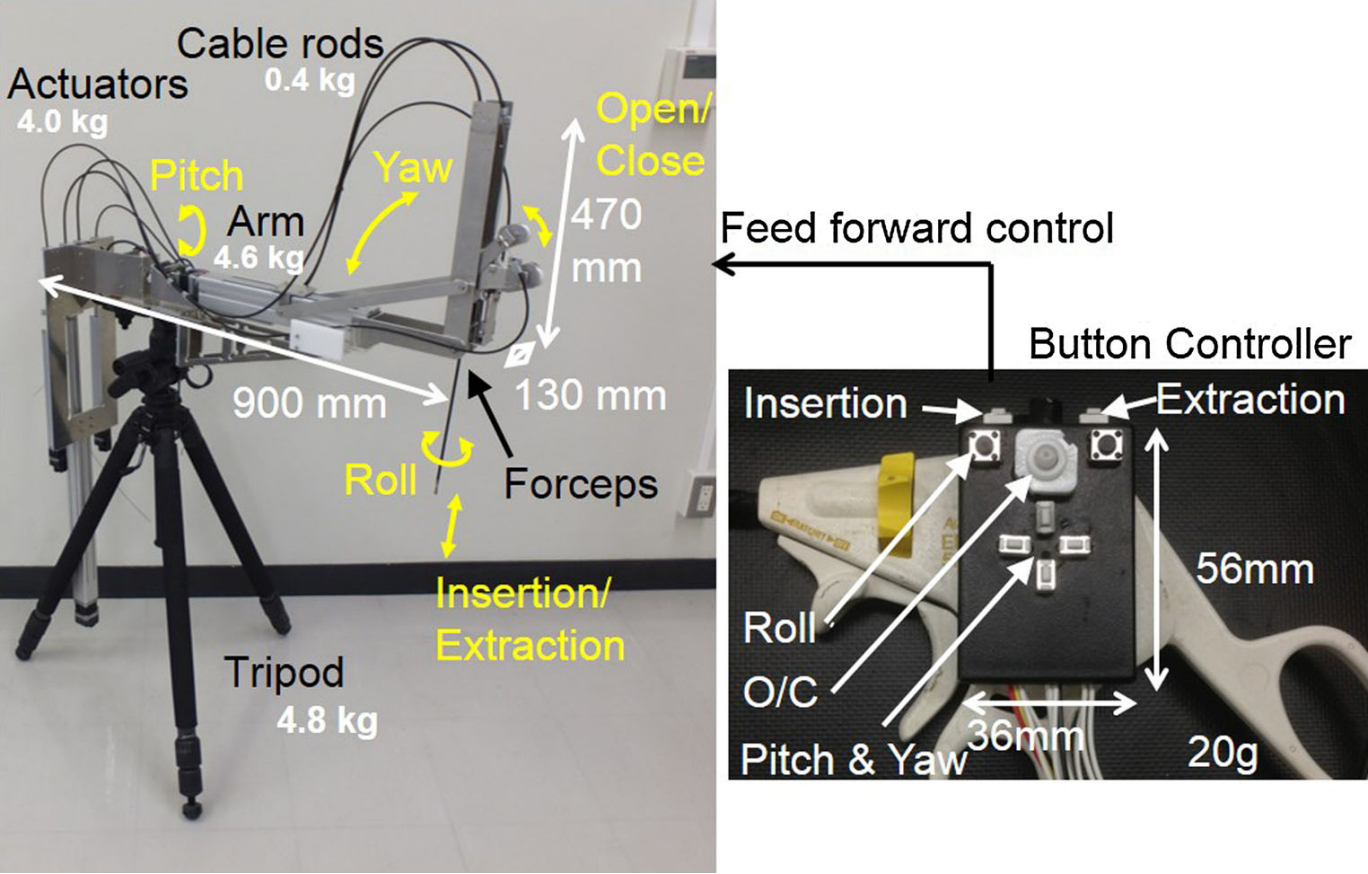
Prototype of mobile LODEM. A commercial forceps attached to the manipulator is controlled using a button controller placed on a handheld forceps. The manipulator can be disassembled into the main arm, the actuators, the cable rods and the tripod. The controller had different shaped switches and a symmetric layout

Figure 3 shows photographs of the prototype LODEM and the handheld control forceps. The operating ranges were $\pm 70^{\circ }$ for the pitch and yaw axes, 0–250 mm for the insertion/extraction axis, $\pm 180^{\circ }$ for the roll axis and 0$^{\circ }$–90$^{\circ }$ for open/close axis. The driving resolution at the tip of a forceps with a length of 250 mm was 0.06 mm for the pitch axis, 0.17 mm for the yaw axis, and 0.01 mm for the insertion/extraction axis, which exceeded the required accuracy of 0.5 mm. The main arm was 470 mm in height, 900 mm in depth, and 130 mm in width, and its mass was 4.6 kg. The cable rod used for the insertion/extraction axis had an inner cable with a diameter of 1.6 mm, and an outer tube with an inner diameter of 2.4 mm and an outer diameter of 5.0 mm. The cable rods used for the roll axis and the open/close axis had an inner cable with a diameter of 1.2 mm, and an outer tube with an inner diameter of 2.0 mm and an outer diameter of 4.0 mm. The length of all the outer tubes was 1,400 mm. Each cable had a mass of 0.1 kg, and the backlash was experimentally determined to be 1.5 mm. The combined mass of the three actuators connected to the cable rods was 4.0 kg, and the tripod had a mass of 4.8 kg. Thus, the total mass was less than 15 kg. All of the actuators were stepper motors, and had sufficient torque to supply a force of over 5 N to the tip of the forceps, based on the friction resistance of the transmission mechanisms.

The button controller attached to the forceps held by the surgeon was the same as that used in the SCARA LODEM prototype [14]. It had different shaped switches and a symmetric layout that made it possible to operate by the right or left hand alone. Its dimensions were 53 $\times \, 36\, \times$ 11 mm, and its mass was 20 g. Commands to control the individual axes were input by pressing the appropriate buttons, and were processed by a motion control board (PCI-7414V, digital input 8 $\upmu$s, pulse rate 6.5 M pulse/s, Interface) installed in a PC, which then output the appropriate signals to the manipulator. The manipulator was feed-forward controlled by a PC system.

### Experimental methods

**Fig. 4:**
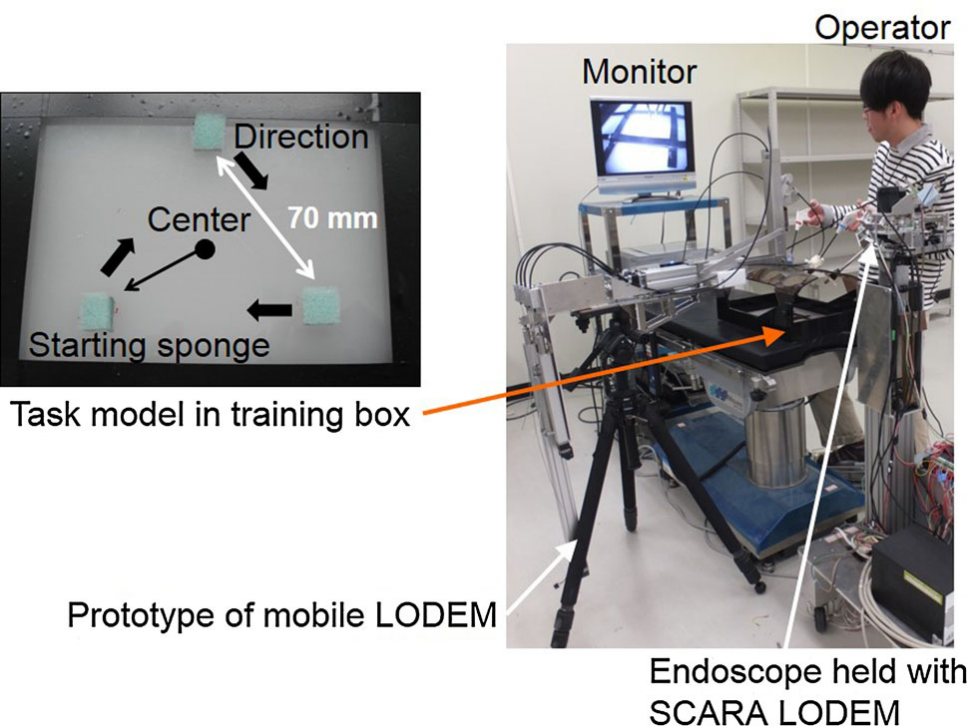
Experimental setup for measuring the time required to complete a task involving a simple model. The model comprised three sponges arranged in a regular triangle with separations of 70 mm. The operator stood on the left-hand side of the table and controlled the prototype mobile LOEDM using the button controller attached to a forceps held in the left hand

To evaluate the prototype, it was used in four different experiments. Experiments 2 and 3 were approved by the ethics committee of Osaka Institute of Technology, and experiment 4 by the ethics committee of Kagawa University. The experiments were as follows:

1. Motion trajectories were measured to confirm the positional accuracy. The trajectory of the tip of the forceps was measured using an optical displacement sensor (accuracy 0.1 mm, OPTOTRAK Certus, NDI) for movements of the pitch, yaw, and insertion/extraction axes. The initial orientation of the manipulator attached to the forceps (diameter 5 mm, ENDO CLINCH II, Covidien) was vertical, and the distance from the tip to the pivot point was 250 mm for the pitch and yaw axes, and 100 mm for the insertion/extraction axis. The tip of the forceps grasped a plate with three optical markers placed on it. The measurement range was $\pm 70^{\circ }$ for the pitch and yaw axes, and 0–10 mm for the insertion/extraction axis. The transmission efficiency of the cable rod was also measured for the insertion/extraction axis under an applied force of 40 N. The radius of curvature of the cable rod was set to 200 mm.
2. The time required to complete a task using a model was evaluated to determine the operability of the device. The well-known procedures to evaluate the operability of the device are laparoscopic skills training model [24]. However, the task of this gold-standard procedure is aimed for the main surgeon to deliver, receive, cut, ligate and knot for organs, it is the suitable procedure for the assistant to reach the forceps to the required position where is grasped and pulled for organs. Instead, a simple triangular model was used, based on the baseball-diamond model used for control of the endoscope-holding robot AESOP [25]. The model was a regular triangular shape formed from three pieces of sponge with distances of 70 mm between them. It was placed in a laparoscopic training box (Endowork-pro II, KARL STOLZ) and was viewed through an endoscope (10 mm diameter, SHINKO KOHKI). The endoscope was positioned at the foot of the surgical table, and the monitor was at the head. The manipulator attached to the forceps was positioned at the right-hand side of the table. The operator stood on the left hand side of the table and controlled the manipulator using the button controller attached to a forceps held in the left hand. The participants were seven endoscope specialists and five engineering students who gave written informed consent. Each time trial involved touching the three pieces of sponge in turn with the tip of the forceps attached to the manipulator, which could be verified by a deformation of the sponge. Pre-trial training involved moving the forceps from the center of the triangle to the starting sponge. A total of five trials were performed for each operator. The experimental setup is shown in Fig. 4. In addition, the time required for each of the engineering students to assemble and disassemble the manipulator was measured.
3. Simulated surgery was performed on a surgically realistic gall bladder model (50128, Limbs and Things) to evaluate the ability of the manipulator to handle objects. The model was placed in the same training box and was viewed through a needle endoscope (diameter 3 mm, NITION). The endoscope, the monitor, the manipulator and the operator were positioned as in the former experiment. The operator was an endoscope specialist and used a scissors in the right hand and the forceps with the button controller in the left hand.
4. Laparoscopic cholecystectomy and proctectomy were performed on a swine in vivo by an endoscope specialist to evaluate the ability of the manipulator to handle real organs. The manipulator attached to the forceps was positioned at the right-hand side of the surgical table. An assistant held the endoscope, and the operator stood on the left-hand side or at the foot of the table. The positions of the assistant and the monitor were changed for each surgical procedure. The operator used an electric scalpel in the right hand and the forceps with the button controller in the left hand.

## Results

**Fig. 5:**
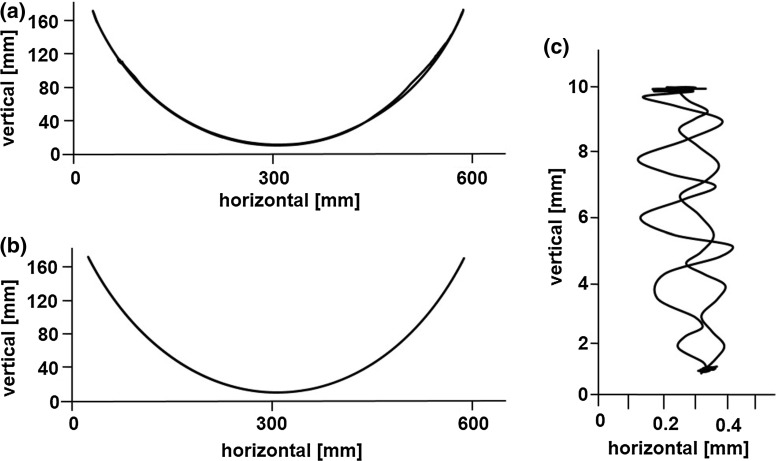
Results of trajectory measurements of forceps tip: **a** pitch axis, **b** yaw axis, **c** insertion/extraction axis. For the pitch, yaw and insertion/extraction axes, the positional accuracy was 0.2, 0.2 and 0.4 mm, respectively

**Fig. 6:**
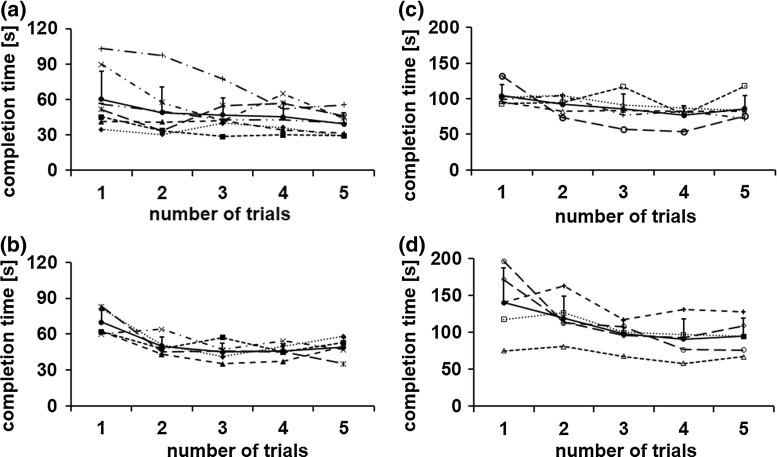
Simple task completion time for: **a** specialists using mobile LODEM, **b** students using mobile LODEM, **c** specialists using previous SCARA LODEM, **d** students using previous SCARA LODEM. The *filled circle symbols* indicate the average time, and the *error bars* represent the standard deviation. *Other symbols* shows the time for each operator

**Fig. 7:**
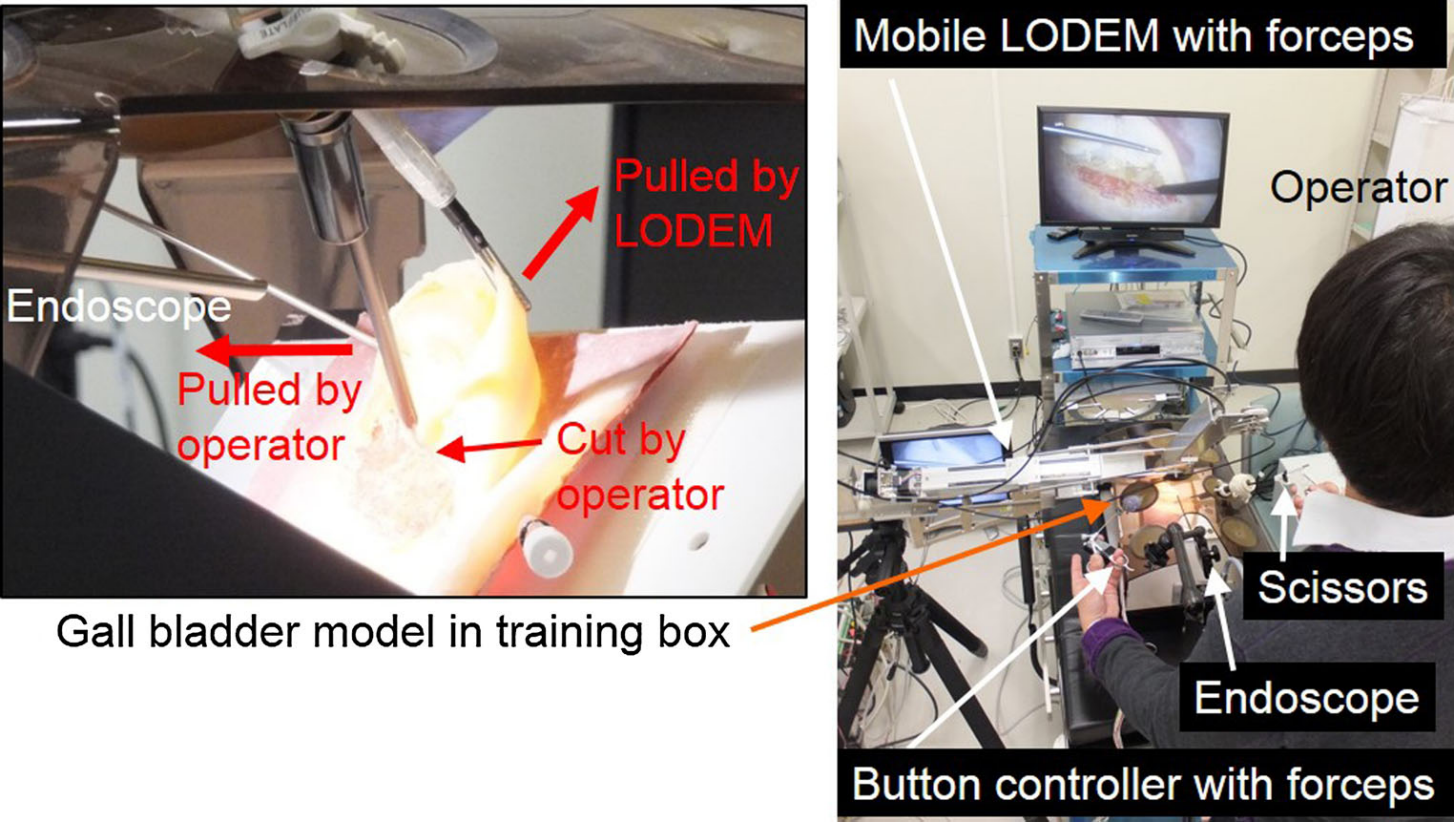
Simulated surgery performed on a surgically realistic gall bladder model by a specialist using the prototype. The model could be grasped and pulled in all directions by the forceps attached to the manipulator. The surgeon could also pull the model organ in opposite directions using the forceps with the button controller in the left hand, and dissect it using the scissors in the right hand

**Fig. 8:**
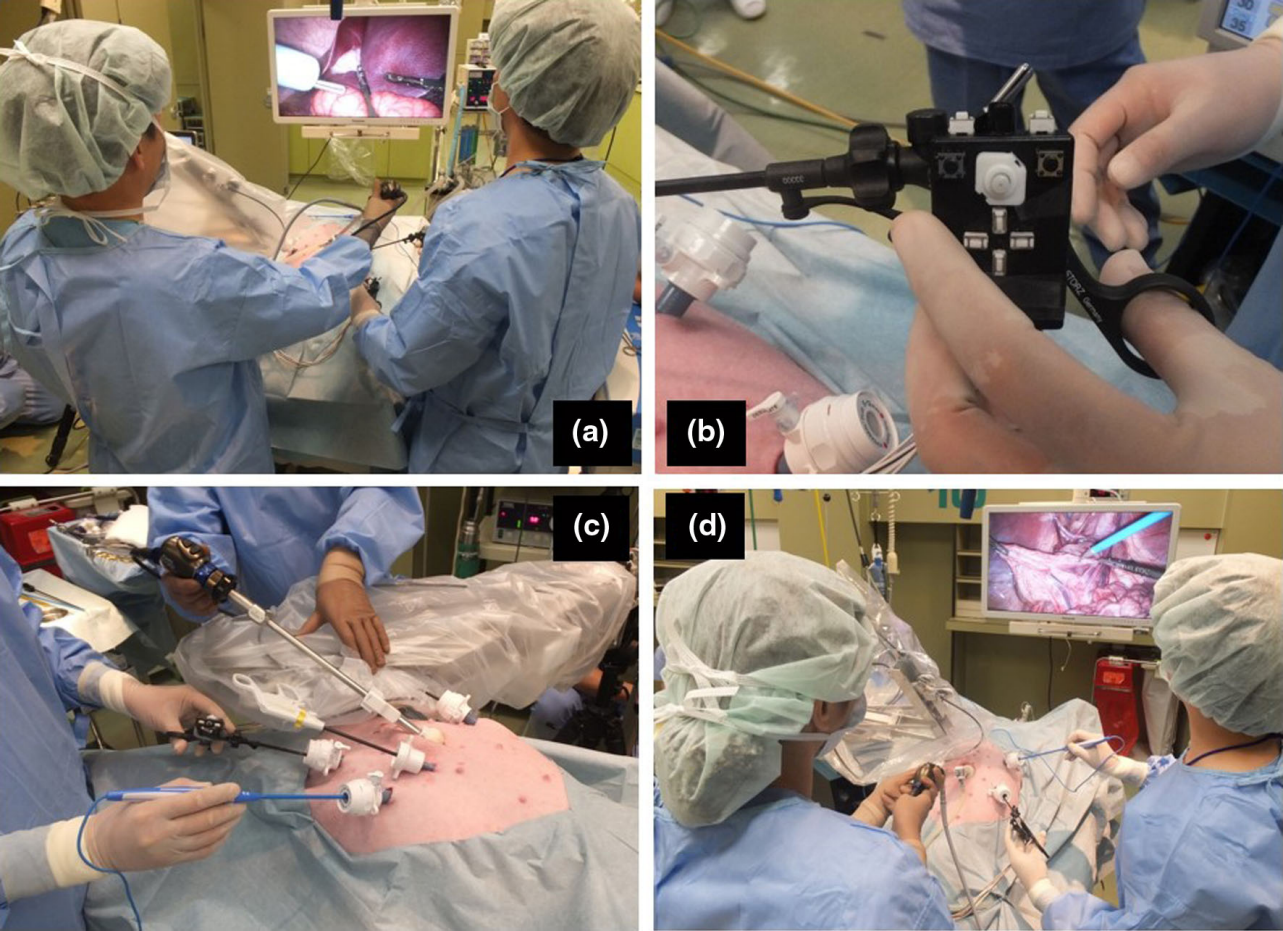
In vivo laparoscopic surgery: **a** grasping and pulling the gall bladder during cholecystectomy, **b** button controller attached to a handheld forceps, **c** horizontal position of manipulator in cholecystectomy, **d** grasping and pulling the colon during proctectomy

The results of the four experiments were as follows:

1. Figure 5 shows the results of the forceps tip trajectory measurements. For the pitch, yaw and insertion/extraction axes, the positional accuracy was 0.2, 0.2, and 0.4 mm, respectively, as seen in Fig. 5a–c. The transmission efficiency of the cable rod for the insertion/extraction axis was 85 % (34 N) for the extraction force and 25 % (10 N) for the insertion force.
2. Figure 6a, b shows the task completion time using the triangular model for the mobile LODEM. As seen in Fig. 6a, from the third trial, the time required by the specialist became constant at about 45 s. For the students, the time became constant at about 45 s, as seen in Fig. 6b. Figure 6c, d shows the time to complete the same task using the previous SCARA LODEM [19]. As seen in Fig. 6c, from the fourth trial, the time required by specialists became constant at about 80 s. For the students, the time became constant at about 90 s, as seen in Fig. 6d. The time required for the students to assemble and disassemble the manipulator was about 8 min.
3. Figure 7 shows the simulated surgical procedure. The gall bladder model could be grasped and pulled in all directions by the forceps attached to the manipulator. The surgeon could also pull the model organ in opposite directions using the forceps in the left hand and dissect it using the scissors in the right hand.
4. Figure 8 shows photographs of the in vivo laparoscopic surgical procedure. In Fig. 8a, the manipulator position is being changed in order to grasp and pull the gall bladder so as to perform the cholecystectomy. The button controller is seen attached to the left-hand forceps in Fig. 8b. Figure 8c shows the manipulator at its maximum pitch angle, making it close to horizontal. Figure 8d shows the manipulator position being changed in order to grasp and pull the colon so as to perform a proctectomy. The forceps attached to the manipulator was made to stop in front of the target organ, grasp and pull it, after the surgeon transferred it to the forceps on the manipulator. The organs could be pulled in various directions using the forceps attached to the manipulator. The forceps held in the left hand could also be used to grasp and pull the organs, and the electric scalpel held in the right hand could be used to dissect the organ. Smooth dissection of the target organ was performed by the specialist. Successful laparoscopic surgery was performed with very little blood loss.

## Discussion

The positional accuracy was 0.5 mm for the previous SCARA LODEM, which was sufficient to use it as a third arm for grasping and manipulating organs [19]. The accuracy of the present mobile LODEM was 0.4 mm, which again is sufficient for handling organs. Since when suturing a vessel, an accuracy of 10 % of its diameter is required, both LODEMs can be used to grasp and pull organs with diameters of 4–5 mm or greater. This makes them suitable for grasping and manipulating the main abdominal organs. For the task shown in Fig. 4, the completion time for endoscope specialists was 80 s using the SCARA LODEM, but only 45 s using the mobile LODEM. In the SCARA LODEM, control of forceps motion in the horizontal and insertion directions is carried out kinematically. In contrast, each axis of the mobile LODEM can be independently mechanically controlled, leading to easier operability. The reason that the specialists were faster than the students was because the students were not used to the endoscopic view. Also, the learning curves for the endoscope specialists became constant from the third trial using the mobile LODEM, but from the forth trial using the SCARA LODEM. These results indicate that the improved mechanism and control in the mobile LODEM led to easier operability. The manipulator could be assembled and disassembled in less than 10 min, making it highly mobile, although the SCARA LODEM could not be disassembled easily. Thus, the mobile LODEM could be assembled and disassembled by medical staff rather than specialist engineers. In both the simulated and in vivo surgical procedures, the manipulator could successfully handle the target organs with the required level of dexterity. Although the size of the gall bladder and colon was only a few tens of millimeters, a surgeon can perform safe endoscopic surgery. The mobile LODEM will allow many types of endoscopic surgery to be carried out safely.

Based on these results, further improvements are being planned so that the mobile LODEM can be put to actual clinical use. First, the positional accuracy for the insertion/extraction axis is worse than the actual resolution of the motor. This is because of buckling of the cable rod and the lack of mechanical stability. Because the fixed actuator had a cantilever shape, vibration was transmitted from the motor to the tip of the forceps. To improve the accuracy so that it matches the resolution of the motor, the actuator is located on the tripod along the slider of the yaw axis. Another problem is that the transmission efficiency of the cable rod in the insertion direction is lower than that in the extraction direction. This is because of bending of the cable rod. The efficiency can be improved by changing the drive direction from push/pull to pull/pull. Finally, it was found that the trocar through the pivot point on the abdominal wall was covered by the manipulator. This leads to a risk of collision with the manipulator if the patient moves. To avoid this, the manipulator should be suitably designed so that there is a separation between it and the patient.

For use in actual surgery, the mobile LODEM must be clean and sterile. After assembling the main arm, actuators, cable rods and tripod, the manipulator can be draped with a sterile cover to keep it clean. The connection between the number of axes and the ability to keep the LODEM clean is three axes: roll, insertion/extraction and open/close. Using separate sterilized attachments made of stainless steel, the sterilized forceps can be attached to the draped manipulator. However, connecting the forceps requires the use of screws and tools. The procedure takes a few minutes and is likely to be annoying for the medical staff. To avoid this, a more ergonomic attachment method has been developed. This uses a bevel gear coupling between a gear inserted in the long axis of the forceps and a gear with a wired pulley for the roll axis, built-in screw clamps in the forceps handle for the insertion/extraction axis, and a gripping chuck inside the finger hole in the forceps handle for the open/close axis. To reduce the setup time, it is likely to be better to assemble the entire mechanical system in the operation room and not use a sterile cover. To improve the usability for sterilization of LODEM, a mechanism to be separated easily into a mechanical drive part following autoclave and an electric drive part that sterilization does not be performed is also under development.

In large city hospitals, doctors can often perform the latest surgical procedures because of the high population density in a city. However, when patients who live in the provinces require such surgery, they must either travel to a city or wait to be operated on in a rural hospital by a visiting doctor. One possible application of endoscopic surgery is carrying out procedures remotely. In this situation, a doctor in a city hospital could operate on a patient in the provinces using a master-slave control manipulator. Although such robotically assisted remote operation is technically possible [20–22], it raises issues of patient safety in the event of emergencies. The local operation using the mobile LODEM is one of the solutions. To improve the safety and effectiveness of LODEM based surgery, a method for measuring the elasticity of an organ using the step-out phenomenon of a stepper motor is currently under development [23].

## Conclusions

We developed a mobile LODEM that can be used by a surgeon as a third arm during endoscopic surgery. It is designed to allow minimally invasive robotically assisted surgery by a doctor working near the patient. The results of the present study indicate that the device is highly promising for such applications. In a future study, the device will be tested for use in single-incision laparoscopic surgery.
